# Descriptive Analysis of Antibiotic-Resistant Patterns of Methicillin-Resistant *Staphylococcus aureus* (MRSA) st398 Isolated from Healthy Swine

**DOI:** 10.3390/ijerph120100611

**Published:** 2015-01-12

**Authors:** Ana Morcillo, Beatriz Castro, Cristobalina Rodríguez-Álvarez, Rossana Abreu, Armando Aguirre-Jaime, Angeles Arias

**Affiliations:** 1 Department of Preventive Medicine and Public Health, University of La Laguna, Canary Islands 38071, Spain; E-Mails: anaimorcillo@gmail.com (A.M.); crrodrig@ull.es (C.R.-Á.); rabreu@ull.es (R.A.); 2 University Hospital of the Canary Islands, San Cristóbal de La Laguna, Tenerife, Canary Islands 38320, Spain; E-Mail: bcastrohdez@yahoo.es; 3 Nuestra Señora de la Candelaria University Hospital, Santa Cruz de Tenerife 38010, Spain; E-Mail: aagujai@gobiernodecanarias.org

**Keywords:** MRSA, SCC*mec*, ST398, swine, antibiotic resistant

## Abstract

*Background*: Livestock-associated methicillin-resistant *Staphylococcus aureus* (MRSA) such as the MRSA ST398 strain has spread all over the World and the most worrying aspect of this fact appears to be its capacity to easily spread to humans. The excessive use of antibiotics has made swine a reservoir of MRSA. The aim of the present study was to determine the antibiotic resistance profile of MRSA samples isolated from healthy swine of the island of Tenerife (Spain). *Methods*: A total of 256 MRSA isolates from swine samples and five MRSA isolates from pig worker samples were investigated for MRSA antibiotic resistant patterns. *Results*: Analysis of the susceptibility status of MRSA pig isolates revealed that 39 isolates were resistant to one antibiotic, 71 isolates were resistant to two antibiotics and 96 isolates were resistant to three or more antibiotics. SCC*mec* typing revealed the presence of types IV and V. Isolates having SCC*mec* IV had an increased resistance to the antimicrobial agents tested than those having SCC*mec* V. We observed significant differences when comparing the most common resistance patterns and SCC*mec* type. *Conclusions*: MRSA isolated from humans showed similar resistance to those isolated from pigs, excepting erythromycin, since all the workers’ isolates were sensitive to this antibiotic. The evolution of new MRSA clones has emphasized the need for infection control practices in animals and humans in close contact.

## 1. Introduction

*Staphylococcus aureus* is a common pathogen and a major public health concern associated with multiple diseases including skin and soft tissue infections, sepsis, osteomyelitis and pneumonia [1,2]. Methicillin-resistant *Staphylococcus aureus* (MRSA) epidemiology has drastically changed in recent years: at first it was a nosocomial pathogen (HA-MRSA) but now infections within the community are becoming more and more common amongst people with no contact with health centres (CO-MRSA). Since 2005, the presence of a distinct clone of MRSA has been reported in a wide variety of animal species and this has been referred to as livestock-associated MRSA (LA-MRSA) [3,4,5,6]. Most animals included in the food chain may be colonized with *S. aureus.* Recently, MRSA strains have been detected in food production animals, such as swine, cattle, chicken and other animals [7,8,9,10], as well as in various types of food products including raw chicken meat, retail pork and beef [11,12,13], milk and dairy products [14,15,16,17,18,19], and fishery products [20]. Previous studies have established that pigs are a reservoir for LA-MRSA ST398 from which humans can be infected and ST398 is currently considered the most prevalent sequence type [21,22] although other molecular types have also been identified [23,24,25]. 

In Spain in 2008, the EFSA study found a prevalence of 46% MRSA in pigs and a prevalence of 46% ST398 linage; non-ST398 MRSA were not detected [26].

The widespread use of antibiotics has led to the emergence of multidrug-resistant strains, making their eradication more difficult. Practices like preventive therapy (mainly of digestive and respiratory disorders), deviations from approved posology (prolonged treatment duration or under-dosing) are common in animal production facilities [27]. This use of antimicrobial drugs in food-producing animals is considered to contribute to the emergence of antimicrobial resistance [28,29]. In its latest report, the European Medicines Agency, 2014 [30], placed Spain as the third largest consumer of antibiotics (1693 tonnes, 21.2% of all antibiotics in the EU) for animal use in food producers.

Van Duijkeren *et al.* [31], found the number of colonized swine in farms applying oral group treatments, often with tetracycline, to be higher compared to farms with no such use of antimicrobials.

Smith *et al.* [32], reported that antibiotics in farms can trigger the emergence of resistant strains and those strains appear in meat, grocery stores and homes, and they can infect people. In 2009, 80% of the antibiotics sold in the United States were used in farms [33]. Although contact with animals seems to be the most important risk factor for human ST398 carriage, meat products may also be a source [34]. In Europe, 46% of total meat consumption corresponds to pork [35] and possible transfer of resistant bacteria via pork meat seems to be inevitable. Therefore, control of antimicrobial resistance in swine should be a priority.

The aim of the present study was to determine the antibiotic resistance profile of MRSA samples isolated from healthy swine and pig farm workers.

## 2. Materials and Methods

### 2.1. Collection of Samples

A cross-sectional prevalence study was conducted. A total of 300 pigs were screened: a randomized selection of 20 animals was taken from 15 wean-to-finishing farms for local consumption. The exploitation systems of swine livestock are of the intensive type, where animals are crammed into farms. The selected farms were those with >250 pigs. Nasal swab samples of healthy pigs, established under veterinarian supervision, from farms all over the island of Tenerife were collected at the Insular Slaughterhouse, between October 2009 and December 2010. Fifty-four nasal swabs of 54 pig workers were included in the study. These workers were from the same livestock farms and from the slaughterhouse [5]. Animals were transported by farm lorry to the Insular Slaughterhouse, where they were kept in separate stables according to their farm of origin and were slaughtered within 12 h. Time of transportation was 1–2 h.

### 2.2. Isolation and Identification of Bacteria

Samples were incubated in brain heart infusion (BHI) with 7% NaCl for 18–24 h at 37 °C. Then 10 µL of the infusion was plated onto MRSA-ID culture plate (bioMérieux^®^, Durham, NC, USA). MRSA colonies were preliminarily identified as characteristically green malachite coloured, round colonies. Isolates were confirmed as *S. aureus* by Gram stain appearance, catalase test and coagulase test agglutination Slidex Staph Plus (bioMérieux^®^). Species identification was confirmed by Vitek^®^ 2 Automated Microbiology System with the ID card GP (bioMérieux^®^). *S. aureus* ATCC 29213 was used as the reference strain. Methicillin resistance was confirmed by testing the presence of penicillin-binding-protein A (PBP2a) (MRSA-screen; Denka Seiken Co™, Tokyo, Japan) and detecting the presence of the gene *mecA* by Real Time PCR (IQ™5; Bio-Rad, Hercules, CA, USA) [5].

### 2.3. Molecular Methods

Molecular typing of MRSA was performed by *ApaI* Pulsed-field gel electrophoresis (PFGE) and *Multilocus Sequence Typing* (MLST). DNA from MRSA isolates was prepared in agarose blocks as described previously. Allelic profiles and sequence types were assigned according to the *S. aureus* MLST database (http://www.mlst.net). The different PFGE types and subtypes obtained were analyzed by SCC*mec* typing as described previously [5].

### 2.4. Antimicrobial Susceptibility Testing

Antimicrobial susceptibility was determined by the broth microdilution method (Vitek^®^ 2 system, bioMérieux^®^) by using AST P588. *S. aureus* ATCC 29213 was used as reference strain. Strains were tested for susceptibility to aminoglucosides: gentamicin (GEN), tobramycin (TOB); betalactamic: bencylpenicillin (PG), oxacillin (OXA); glucopeptides: teicoplanin (TEI), vancomycin (VAN); quinolone: levofloxacin (LVX); lincosamides: clindamycin (CLI); Macrolides: erythromycin (ERY); rifamycin: rifampicin (RF); bacteriostatics trimethoprim-sulfametoxazole (SXT), fusidic acid (FUS); streptogramins: quinupristin–dalfopristin (SYN Q-D); oxazolidinone: linezolid (LNZ), glycylcycline: tigecicline (TGY); phosphate: fosfomycin (FOF); nitrofurane: nitrofurantoin (NIT); monoxycarbolic acid: mupirocine (MUPI). The breakpoints used were the same established by Clinical and Laboratory Standards Institute Guidelines [36]. Multidrug resistant bacteria were defined as bacteria that are resistant to four or more non-β lactam antimicrobials. We compared the resistances to antibiotics, as well as the patterns of resistance according to SCC*mec* IV and SCC*mec* V found in the previous study [5].

### 2.5. Statistical Analysis

Categorical variables were compared with the chi-square test and Fisher’s exact test. All analyses were performed using SPSS 21.00 for Windows (SPSS Inc., Chicago, IL, USA). A *p*-value < 0.05 was considered statistically significant. 

## 3. Results

### 3.1. MRSA Pig Isolates

Antimicrobial susceptibility panel testing was performed on 256 MRSA pig isolates. Resistance levels to antimicrobial for all isolates included: GEN (*n* = 98, 38.3%), TOB (*n* = 101, 39.4%), LVX (*n* = 34, 13.3%), ERY (*n* = 86, 33.6%), CLI (*n* = 129, 50.4%), NIT (*n* = 1, 0.5%) and SXT (*n* = 99, 38.7%).

Analysis of the susceptibility status of MRSA pig isolates revealed that 50 (19.5%) isolates were susceptible to all antibiotics tested, except to betalactamic and 206 included a resistance to other antibiotics: 39 (15.2%) isolates were resistant to one antibiotic, 71 (27.7%) isolates were resistant to two antibiotics, 51 (19.9%) isolates were resistant to three antibiotics and 45 (17.5%) isolates were resistant to four or more non-β-lactam antibiotics. The remaining strains (*n* = 50) were susceptible to all antibiotics tested, except to β-lactam ones. SCC*mec* typing of our MRSA pig isolates revealed the presence of type IV and type V, and PFGE analysis found 12 different patterns, which all belonged to ST398. Isolates having SCC*mec* type IV had an increased resistance to the antimicrobial agents tested than those having SCC*mec* type V (*p* < 0.001). Only levofloxacin isolates with SCC*mec* type V presented a higher resistance. No significant differences were observed between type of SCC*mec* and erythromycin and clindamycin (Table 1).

**Table 1:** Statistical significanceof antimicrobial resistance according to SCC*mec* type detected*.*

Antimicrobial	Number Resistant Isolates		Significance(p)
	SCC*mec* Type *IV*	SCC*mec* Type *V*	
Gentamicin	98	3	<0.001
Tobramycin	98	5	<0.001
Trimethoprim-sulfomethoxazole	88	9	<0.001
Levofloxacin	0	21	<0.001
Erythromycin	37	31	Not significant
Clindamycin	69	60	Not significant

Nineteen different susceptibility patterns were obtained for all of the pig isolates of MRSA analyzed and many profiles were found in each farm. Over 37% of the isolates showed multidrug resistance, 19.5% (50 strains) were susceptible to all tested antibiotics. The resistance profiles found in MRSA pig isolates and associated SCC*mec* types are shown in Table 2.

**Table 2:** Resistance patterns of MRSA isolates.

Resistance Pattern	Pigs		Humans	
	Number	Percentage (%)	Number	Percentage (%)
PG + OXA	50	19.5	2	40
PG + OXA + ERY + CLI	44	17.2	-	-
PG + OXA + GEN + TOB + SXT	41	16.0	1	20
PG + OXA + GEN + TOB + ERY + CLI + SXT	34	13.3	-	-
PG + OXA + LVX + CLI	19	7.4	1	20
PG + OXA + LVX	13	5.1	-	-
PG + OXA + CLI	13	5.1	-	-
PG + OXA + SXT	11	4.3	-	-
PG + OXA + GEN + TOB	8	3.1	-	-
PG + OXA + GEN + TOB + CLI + SXT	8	3.1	1	20
PG + OXA + ERY + CLI + SXT	3	1.2	-	-
PG + OXA + GEN + TOB + CLI	3	1.2	-	-
PG + OXA + TOB + LVX + SXT	2	0.8	-	-
PG + OXA + GEN	1	0.4	-	-
PG + OXA + TOB	1	0.4	-	-
PG + OXA + GEN + TOB + ERY + CLI + FOS + SXT	1	0.4	-	-
PG + OXA + LVX + ERY + CLI	1	0.4	-	-
PG + OXA + GEN + TOB + ERY + CLI + NIT	1	0.4	-	-
PG + OXA + TOB + ERY + CLI	1	0.4	-	-
PG + OXA + GEN + TOB + ERY + CLI	1	0.4	-	-

We observed significant differences when comparing the most common resistance patterns of isolates and SCC*mec* type (*p* < 0.05). Patterns with PG + OXA + ERY + CLI resistance appear more frequently in those isolates having SCC*mec* type V. On the other hand, patterns with PG + OXA + GEN + TOB + SXT resistance and PG + OXA + GEN + TOB + ERY + CLI + SXT resistance are more common in isolates with SCC*mec* type IV. Multidrug resistance was found in 37% of the tested strains. Benzylpenicillin (PG); oxacillin (OXA); levofloxacin (LVX); clindamycin (CLI); erythromycin (ERY); trimethoprim-sulfomethoxazole (SXT); fosfomycin (FOF); nitrofurantoin (NIT); gentamicin (GEN); tobramycin (TOB).

### 3.2. MRSA Human Isolates

Antimicrobial susceptibility panel testing was performed on 5 MRSA workers’ isolates and 4 different antimicrobial resistance patterns were obtained: PG + OXA; PG + OXA + GEN + TOB + SXT; PG + OXA + LVX + CLI and PG + OXA + GEN + TOB + CLI + SXT. We observed that the antimicrobial susceptibility obtained was similar to those obtained in pig isolates excepting erythromycin, since all the workers’ isolates were sensitive to this antibiotic. MRSA strains were SCC*mec* type IV or V, coinciding with those found in pigs, though in the workers SCC*mec* type IV was the majority, while in pigs it was SCC*mec* type V*.*

## 4. Discussion

### 4.1. MRSA Pig Isolates

The problem of the emergence of resistant microorganisms such as MRSA associated with livestock is closely related to the inadequate use of antimicrobial agents in veterinary care [9,37] and international trade of food of animal origin can facilitate the spread of resistant strains. Antibiotics have long been used for treating disease, preventing disease, and improving feed efficiency in conventional livestock and poultry production. Concerns over the spread of antibiotic-resistant genes to human and animal pathogens continue to drive the debate. Alternatives to antibiotics in food-producing animals, such as pre- and probiotics or vaccines, are considered necessary [38]. Intensive livestock productions, where animals are in crowded conditions, are more numerous than expansive livestock productions, where animals are at liberty. Intensive livestock system favours the spread of resistant strains. 

In a study performed by van Duijkeren *et al.* [31] it was demonstrated that the number of swine colonized by MRSA was high in farms that were applying intensive oral treatments, compared with those farms that were not doing it. Nevertheless, in another study conducted by Broens *et al.* [39] they did not find such association. Preventive therapy or the treatment of healthy animals that coexist with unhealthy ones, the use of subdosis or treatments of excessive duration in time can represent a risk factor for the appearance of these strains. As a limitation of this study, we emphasize that in all cases the animals in our study came from intensive production farms, but we did not have access to any information related to their use of antimicrobials.

There are several studies related to the antibiotic susceptibility in MRSA isolated from swine. In our study we obtained a high percentage of resistance to clindamycin (50.4%), tobramycin (39.4%) and gentamycin (38.3%). We also want to highlight the resistance values obtained for trimethoprim-sulfamethoxazole (38.7%) and erythromycin (3.6%). As in other studies, our most represented multi-resistant pattern was the one that included PG + OXA + GEN + TOB + ERY + CLI + SXT, appearing in 13.3% of the cases [9,10,24,25,40,41]. Another important pattern of resistance detected was the combined resistance to erythromycin and clindamycin (17%), a percentage that in our study was lower than in the study of Neeling *et al.* [9], 23%. In the same study they found that 36 % of the strains were resistant to kanamycin, gentamycin and tobramycin, a percentage similar to the one we obtained.

We obtained a high percentage of resistance to trimethoprim-sulfamethoxazole (38.7%), although lower than the 52 % obtained by van Duijkeren *et al.* [31], who affirmed that the pressure of the antimicrobial selection is one of the probable factors that have facilitated the emergence and dispersion of the veterinary MRSA, the most commonly used being tetracycline and trimethoprim-sulfamethoxazole and to which these microorganisms are more resistant. Furthermore, in another Dutch study, Van den Broek *et al.* [37] found that 52% of MRSA were resistant to trimethoprim-sulfamethoxazole, erythromycin and clindamycin. Smith *et al.* [42], in a study of antibiotic susceptibility in MRSA, found that all isolates were susceptible to the majority of the antibiotics tested (trimethoprim-sulfamethoxazole, levofloxacin, vancomycin, aminoglucoside, *etc.*). Nevertheless, as previously mentioned, we found a significant percentage of strains resistant to trimethoprim-sulfamethoxazole, aminoglucoside and levofloxacin. These authors gave lower erythromycin resistance rates than ours (20%), but they found a 87% resistance to clindamycin. Battisti *et al.* [24] obtained higher rates of resistance than ours to trimethoprim-sulfamethoxazole, (68.8%) and erythromycin (60.9%). In the study performed by Osadebe *et al.* [43], 83% of MRSA isolates were resistant to erythromycin, 50% had intermediate resistance to doxycycline and 25% to ciprofloxacin, while all strains were susceptible to linezolid, rifampycin, vancomycin, and trimethoprim-sulfamethoxazole.

All the MRSA strains of the study were ST398 and contained the SCC*mec* that codifies gene *mecA*. When we compared SCC*mec* type IV and V, defined as different short cassettes, with the antibiotic resistance for epidemiological interest, significant differences were observed for gentamycin, tobramycin, levofloxacin and trimethoprim-sulfamethoxazole. MRSA isolates having SCC*mec* type IV had higher resistance rates to gentamycin, tobramycin and trimethoprim-sulfamethoxazole than those with SCC*mec* type V (*p* < 0.005). Nevertheless, in the case of levofloxacin the resistance was predominant in the strains with SCC*mec* type V (*p* = 0.005). In the study conducted by Gómez-Sanz *et al.* [25], they observed that the strains with SCC*mec* type IV presented a higher resistance to gentamycin and tobramycin than those having SCC*mec* type V. We observed the same association. In our study we also observed that the resistance pattern that includes more antibiotics corresponded to those MRSA isolates having SCC*mec* type IV and this difference was significant (*p* = 0.005). Gómez-Sanz *et al.* [25], also indicated that the genotype of multi-resistance is generally associated with the presence of SCC*mec* type IV. In this way we found that resistance pattern PG + OXA + ERY + CLI appeared more frequently in the strains having SCC*mec* type V (*p* = 0.005) and patterns PG + OXA + GEN + TOB + SXT and PG + OXA + GEN + TOB + ERY + CLI + SXT in those having SCC*mec* type IV and these differences were significant (*p* = 0.005). Abreu *et al.* [44] carried out a study on detection of MRSA in black Canarian pigs on the island of Tenerife (Spain); this is an autochthonous porcine race considered to be a descendant from the Mediterranean pig, and also found a high prevalence of colonization (62%), concretely with the ST-398 strain. Resistance pattern PG + OXA + GEN + CLI + SXT was observed in the majority of isolates. [44]. Moreover, another study performed in Spain by Porrero *et al.* [45], comparing carriage of MRSA in Iberian pigs (IP) and Standard white pigs (SWP) found low prevalence and higher susceptibility to antibiotics in IP. The resistance pattern to gentamicin, ciprofloxacin and erythromycin was higher in SWP than in IP.

### 4.2. MRSA Human Isolates

MRSA ST398 has been described as a cause of infections in people occupationally exposed to pigs, by direct or indirect contact and occasionally can be introduced into hospitals as a result of community-acquired human infections. Although it may become part of the endemic flora of the slaughterhouse, the risk of infection to slaughterhouse workers and people handling meat appears to be low. In most cases, colonization with MRSA ST398 in humans is not associated with disease, although clinical cases associated with MRSA ST398 have been reported. MRSA ST398 can be introduced into hospitals via colonized farmers and other people in a region with intensive pig farming. However, it seems that the capacity for dissemination in humans (patient-to-patient transmission) of livestock-origin MRSA is lower as compared to hospital-associated MRSA [26].

Different studies have demonstrated the correlation between the nasal colonization by MRSA with the frequency of contact with pigs apart from the high presence of MRSA strains in farms; many reports comment the colonization of farm workers with ST398 [7,22,46]. The prevalence of MRSA found in our study was less than that found in other countries [21,22,32]. A limitation of the study was the small number of isolates in workers that could not perform the statistical analysis of susceptibility patterns of MRSA isolated from pig and workers’ samples.

In relation to resistance patterns, there are some studies such as Wulf *et al.* [22] who isolated MRSA from 34 veterinarians from a total of 272 attending a convention in the Netherlands; after antibiotic sensitivity tests, 58% of the isolates were resistant to five or more types of antibiotics. They associated these results to antibiotic consumption by livestock. They found strains highly resistant to tetracycline (70%), trimethoprim-sulfamethoxazole (SXT (48%) and gentamicin (20%). They also showed that 4/31 resistant to ciprofloxacin isolates corresponded to two veterinarians from Italy and two veterinarians from Spain. Smith *et al.* [42] indicated that none of the 9 ST398 MRSA isolates from humans were resistant to erythromycin or quinupristin-dalfopristin, but one of them (11%) was resistant to clindamycin. The susceptibility patterns obtained in this study were PG + OXA + GEN + TOB + CLI + SXT, PG + OXA + GEN + TOB + SXT and PG + OXA + CLI + LVX (Table 2); the other two isolates were resistant only to β-lactams. Van den Broek *et al.* [37] obtained higher rates of antimicrobial resistance than in our study. He compared the prevalence of MRSA in population with and without pig contact, indicating that all MRSA ST398 isolates in humans showed a high resistance to trimethoprim-sulfamethoxazole (SXT), erythromycin and clindamycin (52%).

## 5. Conclusions

The findings from this study support some of the results from other studies. All MRSA strains were isolated from healthy swine and it should be noted that 37% of the isolates were multidrug resistant, considering that major control program of the use of antibiotics in veterinary care is necessary in order to detect any expansion or increase of new antibiotic resistance as this could be a public health problem because of their possible transmission to the community. Systematic surveillance and monitoring of MRSA in humans and food producing animals is recommended.
